# Lipoprotein(a): the underutilized risk factor for cardiovascular disease

**DOI:** 10.21542/gcsp.2019.11

**Published:** 2019-09-20

**Authors:** TZ Khan, SR Bornstein, M Barbir

**Affiliations:** 1 Harefield Hospital, Royal Brompton & Harefield NHS Foundation Trust Hospital, Hill End Road, Harefield UB9 6JH, United Kingdom; 2 University Hospital Carl Gustav Carus, Fetscher Street 74, Dresden 01307, Germany

## Abstract

Raised lipoprotein(a) [Lp(a)] is an important independent cardiovascular risk factor and predictor of adverse outcomes. Challenges remain with regards to the screening, diagnosis and management of this condition. Although further prospective randomised controlled data is required, there is growing evidence suggesting that lowering Lp(a) may reduce the risk of cardiovascular events and ameliorate symptoms.

## Introduction

Although lipoprotein(a) [Lp(a)] was first discovered in the 1960s by Berg^1^, the exact physiological role of Lp(a) is still poorly understood. Large scale epidemiological studies have demonstrated that elevated Lp(a) level (>600 mg/L) is an important independent cardiovascular risk factor and predictor of adverse outcomes in atherosclerotic disease^2,3^. However, the subject of Lp(a) remains a dilemma in contemporary cardiovascular medicine in terms of whom we should screen and subsequently treat, the heterogeneity of available assays and ongoing challenges in management. With new therapeutic options on the horizon, we need to establish some clarity on whom we should screen and treat and determine which therapies will offer the best chance of lowering cardiovascular risk amongst those with raised Lp(a).

## The significance of Lp(a)

Large epidemiological studies have erased any doubt that elevated Lp(a) represents a significant independent cardiovascular risk factor. A very large epidemiological multi-centre study on Lp(a) assessed individual records of 126,634 participants in 36 prospective studies^4^.

The association of Lp(a) with coronary heart disease (CHD) was broadly continuous in shape and curvilinear, with no evidence of a threshold. The relative risk of CHD per 3.5-fold higher Lp(a) level adjusted for age and sex only was 1.16 and 1.13 (95% CI [1.09–1.18]) following further adjustment for systolic blood pressure, smoking, history of diabetes and total cholesterol^4^. This suggests that the association is only minimally confounded by conventional risk factors.

Similarly, a recent prospective study found that the Lp(a)/CHD risk association did not depend on levels of other CVD risk factors, including LDL cholesterol levels^5^. Accordingly, amongst 18,720 participants from the European Prospective Investigation of Cancer (EPIC)-Norfolk cohort, Lp(a) levels were associated with future peripheral artery disease (PAD) and coronary artery disease (CAD) events^6^, and the association between Lp(a) and cardiovascular disease was not modified by LDL cholesterol levels.

More recently Khera et al. demonstrated that in a cohort of 9612 JUPITER participants with low LDL cholesterol and elevated hsCRP, Lp(a) was a significant determinant of residual cardiovascular risk^7^. This implies that despite aggressive LDL cholesterol reduction, raised Lp(a) still confers residual risk which deserves further attention. Further prospective studies will help to assess the impact of specifically lowering Lp(a) concentrations to potentially reduce this residual risk. Raised Lp(a) is also prevalent amongst patient with refractory angina and was recently demonstrated in 60% of a cohort of patients with refractory angina^8^, suggesting a potential causal role in this challenging condition.

**Figure 1.**
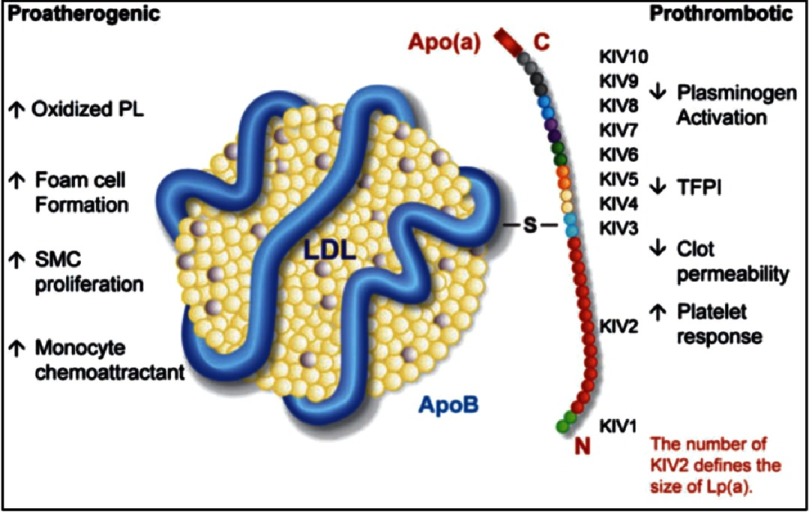
The structure and proatherogenic and prothrombotic actions of lipoprotein(a).

## Measurement of Lp(a)

The heterogeneity of available Lp(a) assays remains a source of confusion amongst clinicians as well as a confounding factor in clinical research studies. Lp(a) is a genetically determined plasma lipoprotein consisting of a cholesterol-rich LDL particle with one molecule of apolipoprotein B100 and an additional protein, apolipoprotein(a), attached via a disulphide bond, which is proatherogenic and prothrombotic via numerous mechanisms (Figure 1^9^). It has been shown that apolipoprotein(a) [apo(a)] size heterogeneity affects the outcome of the immunochemical methods that are still abundantly used to measure Lp(a)^10^; and that the inaccuracy of Lp(a) values determined by methods sensitive to apo(a) size significantly affects the assessment of individual risk status for coronary artery disease^10^.

The first World Health Organization/International Federation of Clinical Chemistry and Laboratory Medicine (WHO/IFCC) International Reference Reagent for Lp(a) immunoassays was approved in 2004 with an accuracy-based value assigned in molar concentration (nmol/L) of Lp(a) particles^11^. A monoclonal enzyme-linked immunosorbent assay (ELISA) method that is unaffected by apo(a) size variation was designated as the internationally recommended reference method^11^. However, much work remains in terms of establishing widespread standardised use of the reference method across centres worldwide.

## Whom should we test?

The 2016 ESC guidelines for the management of dyslipidaemias^12^ recommends Lp(a) screening in individuals with:

• Premature CVD (<55 years in men and <65 years women).
• Familial hypercholesterolemia.
• A family history of premature CVD and/or elevated Lp(a).
• Recurrent CVD despite optimal statin treatment.
• ≥5% 10-year risk of fatal CVD according to SCORE.
• Subjects with borderline risk for reclassification of risk.

The uptake of screening for Lp(a) and awareness of its significance as an important cardiovascular risk factor remains poor, and the vast majority of cardiac and primary care centres are not currently following these recommendations, leaving a substantial proportion of affected individuals undiagnosed. Furthermore, the fact that Lp(a) levels are co-dominantly inherited^13^, raises a plethora of complex questions surrounding cascade screening and thereby the contentious issue of paediatric testing and the optimal timing for screening.

**Figure 2.**
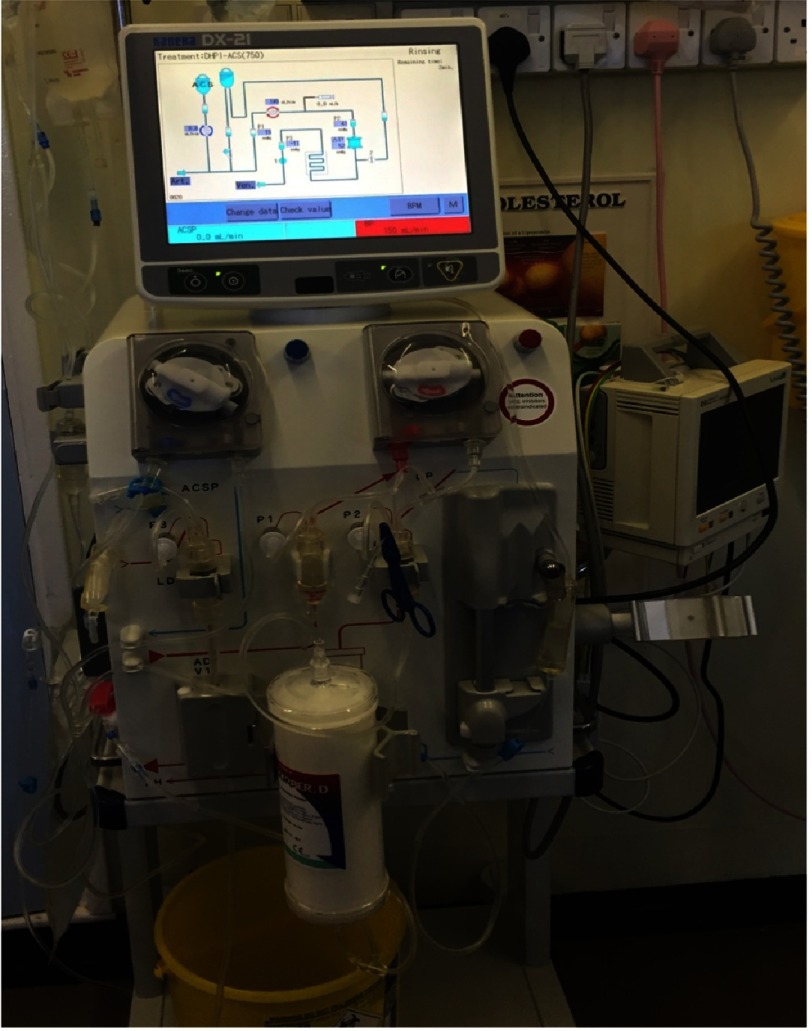
A whole blood lipoprotein apheresis system.

## Challenges in current management

One may argue that we should only screen for a condition if we are prepared to treat it and certainly challenges remain in terms of available therapies for raised Lp(a). To date, therapeutic options to lower Lp(a) levels have been limited. Statins do not have any significant impact on Lp(a) levels^14^, either because Lp(a) is catabolized by alternative mechanisms to the LDL receptor pathway, or because Lp(a) transports PCSK9, which counteracts the statin effect. Nicotinic acid lowers Lp(a) levels by about 25%, most likely by interfering with its production^15^. However, despite its positive effects on Lp(a) as well as the conventional lipoprotein traits LDL-C, HDLC, and triglycerides; nicotinic acid was not found to reduce cardiovascular risk over and above statin therapy^16^, hence it is not recommended for preventive treatment.

Inhibitors of PCSK9, which are increasingly used for statin resistant hypercholesterolaemia, were found to lower Lp(a) levels by approximately 25%^17^. However, it remains unknown whether this effect contributes to the lowering of cardiovascular risk by PCSK9 inhibitors; which could be resolved by conducting a large phase III clinical outcomes trial assessing the impact of PCSK9 inhibitors in patients with exclusively raised Lp(a), given most patients assessed in studies to date had concomitant raised levels of LDL cholesterol^17^. Currently lipoprotein apheresis, a lipid-lowering extracorporeal treatment by which atherogenic ApoB100-containing lipoproteins including Lp(a) and LDL cholesterol are removed from blood or plasma (Figure 2); remains the most effective available means of lowering Lp(a) levels^18^. Lipoprotein apheresis can acutely decrease Lp(a) by approximately 60–75%^19^ and is increasingly recognised as the gold standard of treatment for elevated Lp(a). However, some consider lipoprotein apheresis to be cumbersome and prohibitive in terms of the cost, training and infrastructure required to deliver the service.

## Establishing the impact of treating raised Lp(a)

There remains a paucity of prospective randomised controlled trial data which aims to examine the impact of aggressively treating raised Lp(a). An observational cohort study demonstrated that patients who commenced lipoprotein apheresis because of elevated Lp(a) and progressive cardiovascular disease showed a reduction in major adverse coronary events from 0.41 to 0.09 per year^20^. Similar findings were observed in another retrospective observational study^21^. In a single-blinded randomised controlled trial in patients with refractory angina and raised lipoprotein(a), we recently demonstrated that lipoprotein apheresis led to significant improvement in myocardial perfusion, atheroma burden, exercise capacity, angina symptoms and quality of life^22^. This suggests that lipoprotein apheresis should be considered as a therapeutic option in patients with refractory angina and raised Lp(a), especially given the relatively high prevalence of raised Lp(a) amongst patients with this problematic condition^8^, for whom treatment options remain limited. However, more randomised controlled prospective trial data is required to globally convince clinicians to aggressively treat raised Lp(a).

## New emerging treatments and hope for the future

Most recently, antisense technology offers hope and has been used to develop a specific therapy to lower Lp(a) plasma levels^23^. The most recent generation of antisense oligonucleotides directed against apo(a) mRNA lowers Lp(a) levels dose dependently, by 30 to ≥80% in a single-dose approach, and by 60% to ≥90% in a multi-dose approach^24^. However, further phase II and III trial data is required to establish the safety of this treatment and the impact on cardiovascular events before it can be established for widespread use.

## Conclusion

To conclude, although there is little doubt that raised Lp(a) is an important independent cardiovascular risk factor and predictor of adverse outcomes, challenges remain in terms of screening, testing methods, treatment decisions and access to effective therapy. Lipoprotein apheresis, albeit a cost intensive procedure, is increasingly recognised as the most efficient treatment and the gold standard for management of elevated Lp(a) and there is growing evidence to suggest that it should be utilised more widely to reduce the risk of cardiovascular events and ameliorate symptoms. Although emerging therapies offer hope for the future, further prospective randomised controlled data is required to confirm whether there should be global implementation of aggressive Lp(a) lowering therapy.

## Competing interests

All authors confirm that they have no competing interests to declare.
